# Non-Contact Respiratory Rate Estimation in Real-Time With Modified Joint Unscented Kalman Filter

**DOI:** 10.1109/ACCESS.2020.2998117

**Published:** 2020-05-28

**Authors:** Can Uysal, Altan Onat, Tansu Filik

**Affiliations:** 1 Electrical and Electronics Engineering DepartmentEskisehir Technical University26555EskisehirTurkey; 2 School of Engineering, Stephenson BuildingNewcastle University 5994 Newcastle upon TyneNE1 7RUU.K.

**Keywords:** Unscented Kalman filter, joint unscented Kalman filter, respiratory rate tracking, device-free, vital signs, health monitoring, radio signals

## Abstract

It can be life-saving to monitor the respiratory rate (RR) even for healthy people in real-time. It is reported that the infected people with coronavirus disease 2019 (COVID-19), generally develop mild respiratory symptoms in the early stage. It will be more important to continuously monitor the RR of people in nursing homes and houses with a non-contact method. Conventional, contact-based, methods are not suitable for long-term health monitoring especially in-home care services. The potentials of wireless radio signals for health care applications, such as fall detection, etc., are examined in literature. In this paper, we focus on a device-free real-time RR monitoring system using wireless signals. In our recent study, we proposed a non-contact RR monitoring system with a batch processing (delayed) estimation method. In this paper, for real-time monitoring, we modify the standard joint unscented Kalman filter (JUKF) method for this new and time-critical problem. Due to the nonlinear structure of the RR estimation problem with respect to the measurements, a novel modification is proposed to transform measurement errors into parameter errors by using the hyperbolic tangent function. It is shown in the experiments conducted with the real measurements taken using healthy volunteers that the proposed modified joint unscented Kalman filter (ModJUKF) method achieves the highest accuracy according to the windowing-based methods in the time-varying RR scenario. It is also shown that the ModJUKF not only reduces the computational complexity approximately 8.54% but also improves the accuracy 36.7% with respect to the standard JUKF method.

## I. Introduction

Elderly monitoring systems are becoming widespread and continuously monitoring of respiratory rate (RR), especially for those living alone and suffering from respiratory diseases is vital [1]. Especially, the infection with severe acute respiratory syndrome (SARS), Middle East respiratory syndrome (MERS) and COVID-19 can cause a severe viral respiratory illness. For the conventional RR monitoring methods such as capnography or photoplethysmography, the physical contact with the human body is required [2]. These conventional contact-based methods are not suitable especially for long-term monitoring at home or nursing homes since they restrict people’s movements and prevent them from performing their daily activities. Non-contact (device-free) RR monitoring methods are developed for eliminating the disadvantages of contact-based systems especially in-home care services.

In recent years since wireless radio communications signals are ubiquitous in indoors, radio-frequency (RF)-based monitoring methods (such as fall detection [3], [4], indoor localization [5], etc.) become increasingly popular. These RF based methods are not requiring day-light and line-of-sight propagation, and annihilating users’ privacy concerns [6].

It is observed that the chest movement in the area of interest during breathing, which is a slow and periodic movement, can be sensed using radio waves. In this context, the radar-based methods are existed in literature [7]–[9]. However, these methods have high computational burden since they need time and phase synchronization between the radio transmitter and the receiver. They also require costly equipment and specific antennas. On the other hand, the ambient RF signals which are commonly exist in indoors such as WiFi, can also be used for RR estimation. These methods can be divided into two groups depending on whether they use the received signal strength (RSS) [10], [11] or the fine-grained channel state information (CSI) parameters [12]–[15] of the ambient WiFi signals for monitoring. In addition to these wireless sensing systems, other non-contact methods which are using different technologies such as audio [16] and visual [17] are also investigated in literature.

In these methods the RR is assumed as a deterministic unknown constant number and batch processing (windowed) methods are widely used. It is shown that in a steady indoor environment, the averaged RR rate is successfully estimated using RF signals. These methods can be grouped as FFT-based methods (such as periodogram) [10], [13]–[15], [18], [19], and high resolution subspace techniques [12], [20]–[22]. In the periodogram method, the frequency resolution and accuracy completely depend on the duration of the collected data window. The RR is a time-varying parameter, assuming it is constant over a long period of time, increases the estimation latency and this can be a critical problem in emergencies. Although we showed in our recent studies that the subspace techniques, estimation of signal parameters by rotational invariance technique (ESPRIT) and multiple signal classification (MUSIC), for the RR estimation with a reduced latency and improved accuracy according to the periodogram methods [21], [22], a real-time RR tracking is still an important requirement. In [23], Kalman Filtering (KF) approach for RR estimation is investigated with a limited accuracy.

In this paper, the nonlinear model-based KF approach which is capable of estimating and tracking the RR in real-time with a low steady-state error is proposed. The KF is an optimal filter for linear systems and widely used in control, robotics, navigation systems, target tracking, and communication systems [24]. But, most of the real systems can not be modelled as linear systems such as biomedical systems [25], [26], neural networks [27], and smart grids [28], [29]. For these inherent nonlinear systems, extended Kalman Filter (EKF), which provides an approximation to optimal estimation, is developed [30], [31]. In EKF, since the first-order linearization is applied to the nonlinear model, the mean and covariance of the approximations highly deviate from their actual values. This may cause poor estimation performance and even divergence of the filter. To overcome this problem, the Unscented Kalman Filter (UKF) is developed [32], [33]. It is evaluated in many KF applications that UKF outperforms EKF [34], [35]. In UKF, true mean and covariance values are captured by using the deterministic sigma points. The UKF and its variants are successfully used in various fields such as attitude estimation [36], target tracking [37], power systems [38], [39], vehicle navigation [40]–[42].

**FIGURE 1.**
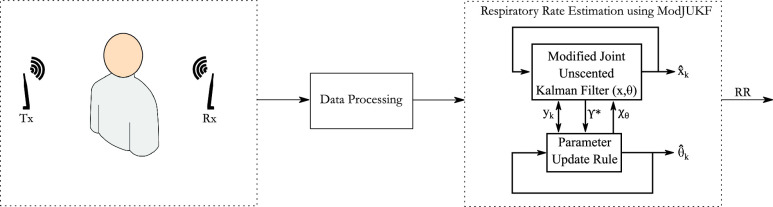
The overview of the proposed non-contact real-time respiratory rate tracking system using ModJUKF method.

The problem of estimating the vital signs such as RR is difficult due to the inherent nonlinearity of the problem. It is required to find the most appropriate KF approach for the RR monitoring problem. There are other types of KF besides UKF for nonlinear models such as particle KF [43], cubature KF [44]–[46], and ensemble KF [47]. All these types of KFs are proposed to solve the problems especially when the model’s state and/or parameter dimensionality is high. Similarly, the other variants of UKF such as iterative one in [48], provides better state and covariance estimation when the dimensionality is high and the system is too complicated. Some other recent versions of UKF in [49]–[51] are proposed to give more robust results when there are outliers, incorrect states and measurements. But, in our case since the RR state-space model can be formulated with two states and one parameter, the standard UKF and the modification of standard joint UKF (JUKF), which is widely used for parameter estimation [52]–[56], are considered. In [57], it is shown that the modification for JUKF, decreases the computational complexity which is called as modified joint unscented Kalman filter (MJUKF). Both JUKF and MJUKF are based on unscented transformation [57]–[60]. In JUKF, the parameters are combined with the state vector and estimated together with the states [61]. Thus, the computational complexity of the JUKF increases depending on the number of parameters. In MJUKF, the parameters are separated from the state-space and updated based on the transformation of errors between measurements and transformed sigma points into parameter errors. Thus, MJUKF reduces the computational complexity significantly according to the JUKF while parameter estimates converge. However, it is clear that the modification proposed in [57] works for nonlinear systems in which a linear transformation between measurements and parameters is achieved. Due to the nonlinear structure of the parameter estimation problem with respect to the measurement hereby, a new and novel modification which is sketched in Fig. 1 is proposed and presented in this paper. A hyperbolic tangent function (i.e. $\tanh $) is firstly considered to transform measurement errors into parameter errors. Similar functions (e.g. hyperbolic sine, sigmoid, rectified linear unit function etc.) are generally used as activation functions in artificial neural network concept, [62], [63]. As stated in [63], such activation functions have two critical drawbacks, firstly outputs can be limited to a finite range, and secondly, they provides non-linearity for any function. Depending on the noise level of the measurements and with appropriate selection of the filter parameters, ModJUKF can surpass JUKF in terms of accuracy [57].

In the real-time RR estimation problem which is sketched in Fig. 1, the first critical issue for the system performance is the considered signal model. We utilize the third-order state-space model for signal representation and this model is also considered for frequency tracking in previous studies [64], [65]. The proposed system uses the amplitude of the received unmodulated carrier wave (CW) signals captured by low-cost software defined radio (SDR) modules. Continuous CW signals provide fine-grained amplitude information which does not have the disadvantages of RSS parameter. However, the proposed KF approach is not only limited to this type of signal but can also be applied to any wireless radio signals. We collected signals from healthy volunteers to obtain performances of the proposed real-time monitoring method. It is shown in various experiments that the proposed ModJUKF method outperforms both the windowing-based periodogram method commonly used in the respiratory rate monitoring literature and the super-resolution methods. The main contributions of the paper are summarized as following:

• The proposed system utilizes the continuous CW signals captured by powerful low-cost SDR modules which provide flexibility and versatility to the users.
• Due to the nonlinear structure of the RR estimation problem with respect to the measurement hereby, a new and novel modification is proposed in this paper to transform measurement errors into parameters errors by using the hyperbolic tangent function.
• The proposed new real-time non-contact RR estimation and tracking ModJUKF method reduces the computational complexity and improves the accuracy according to the standard JUKF method. In addition, ModJUKF achieves the highest accuracy among the windowing-based common methods in the time-varying RR scenario.

## II. System Model and Data Processing

In this section, we discuss the model and the third-order state-space formulations of the breathing signal, and data pre-processing procedures.

### A. Breathing Signal Model

The received RF signal, affected by the breathing motion of an adult which is on or near the propagation path between the radio transmitter and the receiver, is called as the breathing signal. In this section, the mathematical formulations of the breathing signal are considered. During breathing, the chest movements of an adult who stands on/near the propagation path of the RF signal variate the magnitude and phase of the received signal. These variations on the received signal’s magnitude can be modeled as a sinusoidal function depending on the periodical change of the chest movements. The magnitude of the received complex-value baseband signal ($r_{k}$) in discrete-time can be written as the following,

\begin{equation*} |r_{k}|=\mu _{k} + x_{k} + u_{k}, \tag{1}\end{equation*}

where $k$ is discrete time index, $\mu _{k}$ is the time-varying DC component (average) value of the received signal, $u_{k}$ is assumed as an additive noise. $x_{k}$ is the waveform due to motion and in this problem it is a breathing signal due to periodic chest movements,

\begin{equation*} x_{k}= A \sin (2\pi f_{R_{k}}k{T_{s}}+\phi), \tag{2}\end{equation*}

where $A$ and $\phi $ denote constant amplitude and phase of the sinusoidal signal, respectively. $T_{s}$ is the sampling period. $f_{R_{k}}$ which is time-varying frequency is the RR of an adult who stands on the propagation path of the radio signal as shown in Fig. 1.

### B. State-Space Formulation

The state-space equations are defined as,

\begin{align*} \mathbf {x}_{k}=&\mathbf {f}(\mathbf {x}_{k-1})+\mathbf {w}_{k} \tag{3}\\ \psi _{k}=&g(\mathbf {x}_{k})+v_{k} \tag{4}\end{align*}

where (3) and (4) are state and measurement models, with nonlinear $\mathbf {f}$ and $g$ functions, respectively. $\mathbf {w}_{k} \approx N(0,\mathbf {Q})$ and $v_{k} \approx N(0,R)$ are uncorrelated noise on states and measurement, respectively. $\mathbf {Q}$ and $R$ are the state and measurement noise covariances, respectively.

The respiratory frequency (rate) of breathing signal in (2) is time-varying and can be called as *instantaneous frequency* [65] and rewritten as,

\begin{equation*} x_{1,k}= A \sin (\Phi _{k}+\phi). \tag{5}\end{equation*}

where third–order state space representation of the signal is given as a rotating vector in Cartesian plane and projections of this rotating vector is given as the states $x_{1,k}$ and $x_{2,k}$, and its angular velocity equals to the instantaneous frequency $x_{3,k}=\Omega _{k}$. The constant frequency case is expressed as:

\begin{equation*} \Omega _{k}= \Phi _{k}-\Phi _{k-1}. \tag{6}\end{equation*}

The noise free relations of state model in (3) can be expressed as,

\begin{align*} x_{1,k}=&\cos (x_{3,k-1})x_{1,k-1}-\sin (x_{3,k-1})x_{2,k-1}, \\ x_{2,k}=&\sin (x_{3,k-1})x_{1,k-1}+\cos (x_{3,k-1})x_{2,k-1}, \\ x_{3,k}=&x_{3,k-1}, \tag{7}\end{align*}

and the measurement model in (4) can be explicitly rewritten as,

\begin{equation*} \psi _{k}=x_{1,k}+v_{k}, \tag{8}\end{equation*}

### C. Real-Time Recursive DC Blocking Filter

**FIGURE 2.**
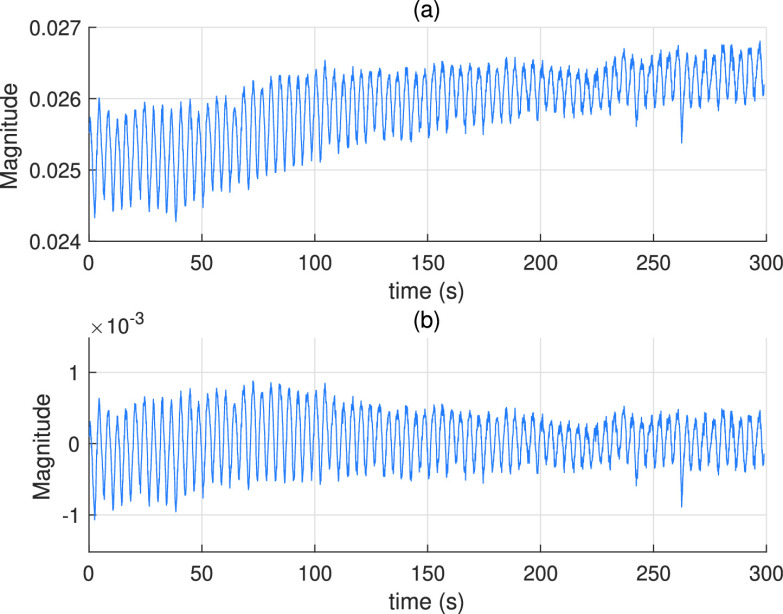
(a) RF signals before passing through the DC blocking filter (b) RF signals after passing through the DC blocking filter.

As shown in Fig. 2. (a) the DC (mean) value of the breathing signal is changing with time. In state-space formulations, the sinusoidal waveform with time-varying frequency in (5) is defined without a DC component. The presence of the DC component disrupts the built-in model and consequently prevents the estimated parameters from converging to the correct value. Thus, DC components do not contain any information about the respiratory rate and must be filtered out from the received signal. To get rid of zero frequency component, a simple method which subtracts the average of the signal from the signal itself can be used. However, this method is not suitable for real-time applications since it requires batch data for computing the average, and also fails since the DC component changes over time. Alternatively, in this study, a recursive DC blocking filter [66], [67] which can operate in real-time is preferred. The transfer function of the system in the Z-domain is written as,

\begin{equation*} H(z)= \frac {1-z^{-1}}{1-pz^{-1}}, \tag{9}\end{equation*}

where $p$ is a coefficient, $0 < p < 1$, and the difference equation of DC blocking filter is given as,

\begin{equation*} y_{k}=\psi _{k}-\psi _{k-1}+p y_{k-1}, \tag{10}\end{equation*}

where $\psi _{k}$ is the measurement data in (8) and $y_{k}$ is the filtered measurement data. The initial value, $y_{0}$, is selected as a very small number close to zero for not creating an offset.

Fig. 2 (a) & (b) show an example of the received RF signal before and after passing through the DC blocking filter, respectively. In real measurements, the received signal may also have a trend not caused by respiratory rate as shown in Fig. 2 (a) due to the time-varying DC components. These trends can cause nonlinearity that adversely affects transient time that is the time for the system to converge the correct parameter. By using DC blocking filter, the trend of the signal alongside the DC component is also eliminated as shown in Fig. 2 (b). UKF assumes white Gaussian noise distributions on states and measurements but for this practical application it can not be always possible. Instead of applying some complex methods in [49]–[51] to overcome this problem, the measurements can be processed by recursive DC blocking filter. So besides getting rid of DC component, unwanted noise components are also eliminated due to the cascaded differentiator/integrator structure [66]. In our case, this filtering improves signal-to-noise ratio (SNR) of the measurements and it also decreases the model and measurement errors of UKF and preventing stochastic stability and divergence problems.

## III. Respiratory Rate Estimation Using ModJUKF

It is reported in [57], [68], [69] that there are two main types of parameter estimation approaches using family of KFs, these are dual and joint filtering. In joint filtering, the parameters are concatenated to state vector and they are estimated by just one filter, whereas in dual filtering, two filters are separately used for the state and parameter estimation. In a joint scheme, since states and parameters are concatenated in an augmented matrix, a cross–covariance between states and parameters are calculated. Nevertheless, in dual filtering such calculation of cross–covariance is missing, [68]. As indicated previously, first modification to JUKF is to create an initial sigma point vector for parameters

\begin{equation*} \boldsymbol {\chi }_{\theta }=\begin{bmatrix} {\hat {\boldsymbol {\theta }}}_{1} & \quad {\hat {\boldsymbol {\theta }}}_{2} & \quad {\dots } & \quad {\hat {\boldsymbol {\theta }}}_{n} \end{bmatrix}^{T}, \quad n=2L+1\tag{11}\end{equation*}

where parameters are *initially* distributed as

\begin{equation*} {\hat {\boldsymbol {\theta }}}_{i}\!=\!\left ({{\hat {\boldsymbol {\theta }}}_{0}\!-\!\mathbf {p}_{\theta }\left ({L+1}\right)}\right)+\mathbf {p}_{\theta } i, \quad i=1, {\dots },2L+1\tag{12}\end{equation*}

where ${\hat {\boldsymbol {\theta }}}_{0}$ is the initial parameter estimate vector, $\mathbf {p}_{\theta }$ contains the initial error covariances of parameters. $i$ is the index of the sigma point of corresponding parameter and $L$ denotes the number of states. In ModJUKF, just like the concatenation of states and parameters into a vector, the corresponding sigma point vectors are also concatenated. Suppose a system has $L$ states and $L_{\theta }$ parameters, the system generates $2(L+L_{\theta })+1$ sigma points for JUKF, whereas for ModJUKF it generates $2L+1$ and it corresponds to the standard state filter (UKF). Clearly, the number of generated sigma points is reduced by $2L_{\theta }$ and in $\frac {(2L_{\theta })}{(2(L+L_{\theta })+1)}\times {100\%}$, if ModJUKF is used for parameter estimation. Nevertheless, computational complexity reduction is less than this percentage due to the separate parameter update rules given in (13) and (14).

The major modification for the JUKF is in the measurement update section, the parameter estimate and sigma point vector update is carried out as

\begin{align*} \boldsymbol {\chi }_{\theta _{i}}=&{\hat {\boldsymbol {\theta }}}^{-}_{k}-\xi T \tanh {\left ({\xi \left ({\mathbf {y}_{k}./ \boldsymbol{\Upsilon }_{i,k|k-1}^{*}-1}\right)}\right)} \\&i=1, {\dots },2L+1 \tag{13}\\ {\hat {\boldsymbol {\theta }}}_{k}=&\frac {1}{2L+1} \sum _{i=0}^{2L} \boldsymbol {\chi }_{\theta _{i}}, \quad i=1, {\dots },2L+1\tag{14}\end{align*}

where ${\hat {\boldsymbol {\theta }}}_{k}$ is the parameter estimate at $k^{th}$ index, $\xi $ is a scaling parameter, $T$ is the transformation matrix with the size of $L_{\theta }\times L_{m}$ which transforms measurement errors of measurement sigma point vector to parameter errors, where $L_{\theta }$ is the number of parameters and $L_{m}$ is the number of measurements. Selection of $T$ is simple and it includes one for each of its element. Scaling parameter $\xi $ is determined based on simulations of the considered system. In RR estimation, there is only one measurement and parameter, so both $L_{\theta }$ and $L_{m}$ are one. Thus, $T$ is considered as one and the parameter $\xi $ selected as 0.025 based on simulations of the system. The selection of $T$ and $\xi $ are the important points of the proposed modification for RR estimation. For the further details of the modification and the selection of $T$ and $\xi $ please refer to [57]. As indicated previously, parameter update rule is changed and a hyperbolic tangent function is considered in this study to include non-linearity between measurements and parameters. Algorithm for the ModJUKF is provided in Algorithm 1.

Algorithm 1 **ModJUKF Algorithm for Additive Noise Case**
1: **Define**Filter Parameters
2: $L,L_{m},L_{\theta }~\triangleright $Size of state,measurement and parameter vector, respectively
3: $\alpha \gets 1~\triangleright $($10^{-4} \leq \alpha \leq 1$)
4: $\kappa \gets 2~\triangleright $(generally $\kappa = 3-L$)
5: $\beta \gets 2$
6: $\lambda \gets \alpha ^{2} (L+\kappa)-L$
7: ${W}_{0}^{(m)}\gets \frac {\lambda }{L+\lambda }$
8: ${W}_{0}^{(c)}\gets \frac {\lambda }{L+\lambda }+1-\alpha ^{2}+\beta $
9: **for** $i \gets \{1, {\dots },2L\}$ **do**
10: $W_{i}^{(m)}\gets W_{i}^{(c)}:=\frac {1}{2(L+\lambda)}$
11: **end for**
12: **End**
13: **function** UKF(${\hat {\mathbf {x}}}$,$\mathbf {P}_{x}$, $\mathbf {p}_{\theta }$)
14: **Initialize**
15: ${\hat {\mathbf {x}}_{0}} \gets \mathbb {E}\left [{\mathbf {x_{0}}}\right]$
16: $\mathbf {P}_{0} \gets \mathbb {E}\left [{(\mathbf {x_{0}}-{\hat {\mathbf {x}}_{0}})(\mathbf {x_{0}}-{\hat {\mathbf {x}}_{0}})^{T}}\right]$
17: ${\hat {\boldsymbol {\theta }}}_{\boldsymbol {0}} \gets \mathbb {E}\left [{\boldsymbol {\theta _{0}}}\right]$
18: $\mathbf {p}_{\theta } \gets \mathbb {E}\left [{(\boldsymbol {\theta _{0}}-{\hat {\boldsymbol {\theta }}}_{\boldsymbol {0}})(\boldsymbol {\theta _{0}}-{\hat {\boldsymbol {\theta }}}_{\boldsymbol {0}})^{T}}\right]$
19: **for** $i \in \{1, {\dots },n=2L+1\}$ **do**
20: ${\hat {\boldsymbol {\theta }}}_{i}\gets \left ({{\hat {\boldsymbol {\theta }}}_{0}-\mathbf {p_{\theta }} \left ({L+1}\right)}\right)+\mathbf {p_{\theta }} i$,
21: **end for**
22: $\boldsymbol {\chi }_{\theta }\gets \begin{bmatrix} {\hat {\boldsymbol {\theta }}}_{1} & {\hat {\boldsymbol {\theta }}}_{2} & {\dots }& {\hat {\boldsymbol {\theta }}}_{n} \end{bmatrix}^{T}$
23: **End**
24: **for** $k \in \{1, {\dots },\infty \}$ **do**
25: **function** Sigma Points(${\hat {\mathbf {x}}}$, $\mathbf {P}_{x}$)
26: $\begin{aligned}\boldsymbol {\chi }_{k-1} \gets &  [{\hat {\mathbf {x}}}_{k-1},{\hat {\mathbf {x}}}_{k-1}+\sqrt {\left ({L+\lambda }\right)\mathbf {P}_{x}},\\ &{\hat {\mathbf {x}}}_{k-1}-\sqrt {\left ({L+\lambda }\right)\mathbf {P}_{x}}]\end{aligned}$
27: **end function**
28: **function** Time Update($\boldsymbol {\chi }_{k-1}$, $\mathbf {P}$, $\mathbf {Q}$, $\boldsymbol {\chi }_{\theta }$)
29: $\boldsymbol {\chi }_{k-1}^{{dagger{ }}}\gets \left [{\boldsymbol {\chi }_{k-1}|\boldsymbol {\chi }_{\theta }}\right]$
30: $\boldsymbol {\chi }_{k|k-1}^{*} \gets f(\boldsymbol {\chi }_{k-1}^{{dagger{ }}})$
31: ${\hat {\mathbf {x}}}_{k}^{-} \gets \sum _{i=0}^{2L} W_{i}^{(m)} \chi _{i,k|k-1}^{*}$
32: $\begin{aligned} \mathbf {P}_{k}^{-} \gets & \sum _{i=0}^{2L} W_{i}^{(c)} (\chi _{i,k|k-1}^{*}-{\hat {\mathbf {x}}}_{k}^{-})\times \\ &(\chi _{i,k|k-1}^{*}-{\hat {\mathbf {x}}}_{k}^{-})^{T}+\mathbf {Q}\end{aligned}$
33: **end function** Measurement and parameter
34: **function** $\begin{aligned}& \text {Measurement and parameter}\\ &\text {Update}\left ({\boldsymbol {\chi }_{k|k-1}^{*},\mathbf {R}}\right) \end{aligned}$
35: $\boldsymbol{\Upsilon }_{k|k-1}^{*} \gets g(\boldsymbol {\chi }_{k|k-1}^{*})$
36: ${\hat {\mathbf {y}}}_{k}^{-} \gets \sum _{i=0}^{2L} W_{i}^{(m)} \Upsilon _{i,k|k-1}^{*}$
37: $\begin{aligned}\mathbf {P}_{{\tilde {\mathbf {y}}}_{k}{\tilde {\mathbf {y}}}_{k}} \gets &\sum _{i=0}^{2L} W_{i}^{(c)} (\Upsilon _{i,k|k-1}^{*}-{\hat {\mathbf {y}}}_{k}^{-})\times \\ &(\Upsilon _{i,k|k-1}^{*}-{\hat {\mathbf {y}}}_{k}^{-})^{T}+\mathbf {R}\end{aligned}$
38: $\begin{aligned}\mathbf {P}_{\mathbf {x}_{k}\mathbf {y}_{k}} \gets & \sum _{i=0}^{2L} W_{i}^{(c)} (\boldsymbol{\chi }_{i,k|k-1}^{*}-{\hat {\mathbf {x}}}_{k}^{-})\times \\ &(\Upsilon _{i,k|k-1}^{*}-{\hat {\mathbf {y}}}_{k}^{-})^{T}\end{aligned}$
39: $\mathbf {K}_{k}\gets \mathbf {P}_{\mathbf {x}_{k}\mathbf {y}_{k}} \mathbf {P}_{{\tilde {\mathbf {y}}}_{k}{\tilde {\mathbf {y}}}_{k}}^{-1}$
40: ${\hat {\mathbf {x}}}_{k} \gets {\hat {\mathbf {x}}}_{k}^{-}+\mathbf {K}_{k}(\mathbf {y}_{k}-{\hat {\mathbf {y}}}_{k}^{-})$
41: $\mathbf {P}_{k}\gets \mathbf {P}_{k}^{-}-\mathbf {K}_{k}\mathbf {P}_{{\tilde {\mathbf {y}}}_{k}{\tilde {\mathbf {y}}}_{k}}K_{k}^{T}$
42: $\boldsymbol {\chi }_{\theta _{i}}\gets {{{\hat {\boldsymbol {\theta }}}_{k}^{-}}}-\xi T\tanh {\left ({\xi \left ({\mathbf {y}_{k}./\boldsymbol{\Upsilon }_{i,k|k-1}^{*}-1}\right)}\right)}$
43: ${\hat {\boldsymbol {\theta }}}_{k} = \frac {1}{2L+1} \sum _{i=0}^{2L} \boldsymbol {\chi }_{\theta _{i}}$
44: **end function**
45: **end for**
46: **end function**

### A. Analysis of ModJUKF

An analysis based on *maximum a-posteriori* estimation perspective (i.e. MAP) can be considered here along with the *statistical linearization* (i.e. SL) to prove that the selection of parameter update rule in (13) can provide an extremum for the *maximum posterior likelihood equation*, and it is used for the theoretical analysis of parameter estimation with sigma point KFs in [68]. Main assumption for the sigma point KFs (e.g. JUKF, DUKF) is that the prior knowledge on system states and parameters are known. To overcome this limitation adaptive versions of unscented Kalman filter are proposed based on *maximum likelihood estimation* (i.e. MLE) [41], and MAP estimation is a regularization of MLE in case of prior statistics. This analysis includes a similar one given in [57], [68] for JUKF and a similar modified JUKF. Conditions, which make the parameter estimate in ModJUKF a MAP estimate, are discussed hereby in comparison with standard JUKF case. Posterior distribution of parameters is

\begin{equation*} {p\left ({\boldsymbol {\theta }_{k}\mid \mathbf {y}_{1:k}}\right)=\frac {p\left ({\mathbf {y}_{k}\mid \boldsymbol {\theta }_{k}}\right)p\left ({\boldsymbol {\theta }_{k}\mid \mathbf {y}_{1:k-1}}\right)}{p\left ({\mathbf {y}_{1:k}}\right)/p\left ({\mathbf {y}_{1:k-1}}\right)}}. \tag{15}\end{equation*}

Previous equation is the result of Bayes rule and the conditional independence of the observation for the current state. The $\boldsymbol {\theta }_{k}$ which maximizes the following equation is selected as MAP parameter estimate in (15),

\begin{equation*} { {\hat {\boldsymbol {\theta }}}_{k}^{MAP}=\arg \max \limits _{{\hat {\boldsymbol {\theta }}}_{k}}\left [{p\left ({\mathbf {y}_{k}\mid \boldsymbol {\theta }_{k}}\right)p\left ({\boldsymbol {\theta }_{k}\mid \mathbf {y}_{1:k-1}}\right)}\right].} \tag{16}\end{equation*}

If the terms inside brackets are written as a negative natural logarithm in (16), it becomes

\begin{equation*} { J\left ({\boldsymbol {\theta }_{k}}\right)=-\ln \left ({p\left ({\mathbf {y}_{k}\mid \boldsymbol {\theta }_{k}}\right)}\right)-\ln \left ({p\left ({\boldsymbol {\theta }_{k}\mid \mathbf {y}_{1:k-1}}\right)}\right).} \tag{17}\end{equation*}

This expression is called as *posterior log–likelihood* function [57], [68], and map parameter estimate is given as ${{\hat {\boldsymbol {\theta }}}_{k}^{MAP}=\arg \min \left [{J\left ({\boldsymbol {\theta }_{k}}\right)}\right].}$

In Kalman filtering framework general assumption is that all noise densities are Gaussian. Probability densities in posterior log–likelihood function [57], [68] can be given as

\begin{align*} {p\left ({\boldsymbol {\theta }_{k}\mid \mathbf {y}_{1:k-1}}\right)}=&{\frac {1}{\sqrt {\left ({2\pi }\right)^{L_{\theta }}\left |{\mathbf {P}^{-}_{{\hat {\boldsymbol {\theta }}}_{k}}}\right |}}\exp \left [{-\dfrac {1}{2}\left ({\boldsymbol {\theta }_{k}-{\hat {\boldsymbol {\theta }}}^{-}_{k}}\right)^{T}}\right.} \\&\times \,{\left.{\mathbf {P}^{-}_{{\hat {\boldsymbol {\theta }}}_{k}}\left ({\boldsymbol {\theta }_{k}-{\hat {\boldsymbol {\theta }}}^{-}_{k}}\right)\vphantom {\left [{-\dfrac {1}{2}\left ({\boldsymbol {\theta }_{k}-{\hat {\boldsymbol {\theta }}}^{-}_{k}}\right)^{T}}\right.}}\right],} \tag{18}\\ {p\left ({\mathbf {y}_{k}\mid \boldsymbol {\theta }_{k}}\right)}=&{\frac {1}{\sqrt {\left ({2\pi }\right)^{L_{m}}\left |{\mathbf {R}_{e}}\right |}}\exp \left [{-\dfrac {1}{2}\left ({\mathbf {y}_{k}\!-\!g\left ({\mathbf {x}_{k},\boldsymbol {\theta }_{k}}\right)}\right)^{T}}\right.} \\&\times \,{\left.{\left ({\mathbf {R}_{e}}\right)^{-1}\left ({\mathbf {y}_{k}\!-\!g\left ({\mathbf {x}_{k},\boldsymbol {\theta }_{k}}\right)}\right)\vphantom {\left [{-\dfrac {1}{2}\left ({\boldsymbol {\theta }_{k}-{\hat {\boldsymbol {\theta }}}^{-}_{k}}\right)^{T}}\right.}}\right],} \tag{19}\end{align*}

where ${{\hat {\boldsymbol {\theta }}}^{-}_{k}}$ is the prior parameter estimate, ${\mathbf {P}^{-}_{{\hat {\boldsymbol {\theta }}}_{k}}}$ is its covariance and ${g\left ({\mathbf {x}_{k},\boldsymbol {\theta }_{k}}\right)}$ is nonlinear observation function given in (4) (i.e. ${\psi _{k}}$), ${\mathbf {R}_{e}}$ is the observation noise covariance. A *statistically linearized* form of this nonlinear function is considered for the analysis of JUKF as a parameter estimator in [68], and it is used to reveal conditions on how both JUKF and ModJUKF provide MAP parameter estimates. By assuming a statistically linearized form, the nonlinear measurement function can be expressed as

\begin{equation*} \mathbf {y}=g\left ({\mathbf {x}_{k},\boldsymbol {\theta }_{k}}\right)\approx \mathbf {A}\boldsymbol {\theta }_{k}+\mathbf {b}. \tag{20}\end{equation*}

The approximation error is

\begin{equation*} \boldsymbol {\epsilon }_{k}\dot {=}g\left ({\mathbf {x}_{k},\boldsymbol {\theta }_{k}}\right)-\mathbf {A}\boldsymbol {\theta }_{k}-\mathbf {b}. \tag{21}\end{equation*}

The main aim here is to find the matrices ${\mathbf {A}}$ and ${\mathbf {b}}$ such that ${J=E\left [{\boldsymbol {\epsilon }_{k}^{T}\mathbf {W}\boldsymbol {\epsilon }_{k}}\right]}$ is minimized for a positive semi–definite matrix ${\mathbf {W}}$. After some manipulation (see [57]), ${\mathbf {A}}$ and ${\mathbf {b}}$ can be found as

\begin{align*} \mathbf {A}=&\mathbf {P}_{\boldsymbol {\theta }_{k}\mathbf {y}_{k}}^{T}\mathbf {P}_{\boldsymbol {\theta }_{k}}^{-1}, \tag{22}\\ \mathbf {b}=&\hat {\mathbf {y}}_{k}-\mathbf {A}{\hat {\boldsymbol {\theta }}}_{k}, \tag{23}\end{align*}

where ${\mathbf {P}_{\boldsymbol {\theta }_{k}}}$ is the parameter covariance matrix and ${\mathbf {P}_{\boldsymbol {\theta }_{k}\mathbf {y}_{k}}}$ is the cross covariance matrix between parameter and measurements. If ${\mathbf {b}}$ is substituted in (21) and it is rearranged, this approximation is given as

\begin{equation*} g\left ({\mathbf {x}_{k},\boldsymbol {\theta }_{k}}\right)= \mathbf {A}\left ({\boldsymbol {\theta }_{k}-{\hat {\boldsymbol {\theta }}}^{-}_{k}}\right)+\hat {\mathbf {y}}^{-}_{k}+\boldsymbol {\epsilon }_{k}, \tag{24}\end{equation*}

where $\boldsymbol {\epsilon }_{k}$ is the *statistical linearization error* and it is assumed as a Gaussian random variable with covariance $\mathbf {P}_{\boldsymbol{\epsilon }}$. Substituting (24) into (4) (here $\psi _{k}$ is the measurement and regarded as $\mathbf {y}_{k}$).

\begin{equation*} \mathbf {y}_{k}= \mathbf {A}\left ({\boldsymbol {\theta }_{k}-{\hat {\boldsymbol {\theta }}}^{-}_{k}}\right)+\hat {\mathbf {y}}^{-}_{k}+\boldsymbol {\epsilon }_{k}+\mathbf {v}_{k}, \tag{25}\end{equation*}

where $\tilde {\mathbf {e}}_{k}=\boldsymbol {\epsilon }_{k}+\mathbf {v}_{k}$ is called as *effective observation noise* and since both $\boldsymbol {\epsilon }_{k}$ and $\mathbf {v}_{k}$ are assumed Gaussian random variable, their sum is also a Gaussian random variable with covariance

\begin{equation*} \mathbf {R}_{\tilde {\mathbf {e}}}=\mathbf {P}_{\boldsymbol{\epsilon }}+\mathbf {R}_{\mathbf {e}}, \tag{26}\end{equation*}

where $\mathbf {R}_{\mathbf {e}}$ is the measurement noise covariance defined as $\mathbf {R}_{\mathbf {e}}=E\left [{\mathbf {v}_{k}\mathbf {v}_{k}^{T}}\right]$ and $\mathbf {v}_{k}$ is expressed in (4). If the alternative form in (25) with the term $\tilde {\mathbf {e}}_{k}=\boldsymbol {\epsilon }_{k}+\mathbf {v}_{k}$ is substituted in (19), then the measurement likelihood density function is presented as

\begin{align*} {p\left ({\mathbf {y}_{k}\mid \boldsymbol {\theta }_{k}}\right)}=&{\frac {1}{\sqrt {\left ({2\pi }\right)^{L_{m}}\left |{\mathbf {R}_{e}}\right |}}} \\&\times \,{\exp \left [{-\dfrac {1}{2}\left ({\mathbf {y}_{k}-\mathbf {A}\left ({\boldsymbol {\theta }_{k}-{\hat {\boldsymbol {\theta }}}^{-}_{k}}\right)-\hat {\mathbf {y}}^{-}_{k}}\right)^{T} }\right.} \\&\times \,{\left.{ \left ({\mathbf {R}_{\tilde {e}}}\right)^{-1}\left ({\mathbf {y}_{k}-\mathbf {A}\left ({\boldsymbol {\theta }_{k}-{\hat {\boldsymbol {\theta }}}^{-}_{k}}\right)-\hat {\mathbf {y}}^{-}_{k}}\right) \vphantom {\left [{\!-\dfrac {1}{2}\left ({\!\mathbf {y}_{k}\!-\!\mathbf {A}\left ({\!\boldsymbol {\theta }_{k}\!-\!{\hat {\boldsymbol {\theta }}}^{-}_{k}\!}\right)-\hat {\mathbf {y}}^{-}_{k}\!}\right)^{T} }\right.}\!\!}\right].}~\qquad ~ \tag{27}\end{align*}

If (27) and (18) are substituted in (17), this posterior log-likelihood function is rewritten as

\begin{align*} {J\left ({\boldsymbol {\theta }_{k}}\right)}=&{\dfrac {1}{2}\left [{\mathbf {y}_{k}-\mathbf {A}\left ({\boldsymbol {\theta }_{k}-{\hat {\boldsymbol {\theta }}}^{-}_{k}}\right)-\hat {\mathbf {y}}^{-}_{k}}\right]^{T}\left ({\mathbf {R}_{\tilde {e}}}\right)^{-1}} \\&\times \,{\left [{\mathbf {y}_{k}-\mathbf {A}\left ({\boldsymbol {\theta }_{k}-{\hat {\boldsymbol {\theta }}}^{-}_{k}}\right)-\hat {\mathbf {y}}^{-}_{k}}\right]} \\&+\,{\dfrac {1}{2}\left ({\boldsymbol {\theta }_{k}-{\hat {\boldsymbol {\theta }}}^{-}_{k}}\right)^{T}\left ({\mathbf {P}^{-}_{{\hat {\boldsymbol {\theta }}}_{k}}}\right)^{-1}\left ({\boldsymbol {\theta }_{k}-{\hat {\boldsymbol {\theta }}}^{-}_{k}}\right).} \quad \tag{28}\end{align*}

MAP parameter estimate is simply found by taking the partial derivative of (28) with respect to $\boldsymbol {\theta }_{k}$ and equating it to zero. MAP parameter estimate providing extremum to (28) is given as (see [57] for derivation)

\begin{align*} {\hat {\boldsymbol {\theta }}}_{k}^{MAP}\!=\! {\hat {\boldsymbol {\theta }}}^{-}_{k}\!+\!\left [{\mathbf {P}_{\boldsymbol {\theta }_{k}}^{-1}+\mathbf {A}^{T}\mathbf {R}_{\tilde {e}}^{-1}\mathbf {A}}\right]^{-1}\mathbf {A}^{T}\mathbf {R}_{\tilde {e}}^{-1}\left [{\left ({\mathbf {y}_{k}\!-\!\hat {\mathbf {y}}^{-}_{k}}\right)}\right]. \!\! \\ \tag{29}\end{align*}

In [68], (29) is given as MAP parameter estimate of JUKF. The Kalman gain in this case is expressed as

\begin{equation*} {\mathbf {K}=\left [{\mathbf {P}_{\boldsymbol {\theta }_{k}}^{-1}+\mathbf {A}^{T}\mathbf {R}_{\tilde {e}}^{-1}\mathbf {A}}\right]^{-1}\mathbf {A}^{T}\mathbf {R}_{\tilde {e}}^{-1},} \tag{30}\end{equation*}

and it results in the parameter update rule for JUKF as

\begin{equation*} {{\hat {\boldsymbol {\theta }}}_{k}^{MAP}={\hat {\boldsymbol {\theta }}}^{-}_{k}+\mathbf {K}\left ({\mathbf {y}_{k}-\hat {\mathbf {y}}^{-}_{k}}\right)}. \tag{31}\end{equation*}

To compare the parameter update rule of ModJUKF and JUKF, writing both rules is a good practice and they are provided as

\begin{align*} \boldsymbol {\chi }_{\theta _{i}}=&{\hat {\boldsymbol {\theta }}}^{-}_{k}-\xi T \tanh {\left ({\xi \left ({\mathbf {y}_{k}./\boldsymbol{\Upsilon }_{i,k|k-1}^{*}-1}\right)}\right)}, \\&{i=1, {\dots },2L+1} \tag{32}\\ {\hat {\boldsymbol {\theta }}}_{k}^{MAP}=&{\hat {\boldsymbol {\theta }}}^{-}_{k}+\left [{\mathbf {P}_{\boldsymbol {\theta }_{k}}^{-1}+\mathbf {A}^{T}\mathbf {R}_{\tilde {e}}^{-1}\mathbf {A}}\right]^{-1}\mathbf {A}^{T}\mathbf {R}_{\tilde {e}}^{-1}\left [{\left ({\mathbf {y}_{k}-\hat {\mathbf {y}}^{-}_{k}}\right)}\right]. \\ \tag{33}\end{align*}

In JUKF, effective observation noise $\mathbf {R}_{\tilde {\mathbf {e}}}$ is considered as constant and by selecting a variable Kalman gain as given in (30), it provides a MAP parameter estimate. Nevertheless, JUKF implicitly calculates covariances between states and parameters. To achieve a MAP parameter estimate, ModJUKF assumes a constant Kalman gain $\xi T$ and a variable $\mathbf {R}_{\tilde {\mathbf {e}}}$. For ModJUKF, it can be expressed as (see [57])

\begin{equation*} {\mathbf {R}_{\tilde {e}}=\left [{-\xi \left ({AA^{T}}\right)^{-1}A\mathbf {P}_{\boldsymbol {\theta }_{k}}^{-1}T\left [{I+\xi AT}\right]^{-1}}\right]^{-1},} \tag{34}\end{equation*}

One may oppose that direct comparison of two equations (32) and (33) is not possible at first glance, since the second terms of the two functions are different. However, it should be noted that as $\hat {\mathbf {y}}^{-}_{k}\rightarrow \mathbf {y}_{k}$ (or $\boldsymbol{\Upsilon }_{i,k|k-1}^{*}\rightarrow \mathbf {y}_{k}$ for ModJUKF) both functions exhibit same behavior. To reveal this fact, the first step is to rewrite (32) as

\begin{align*}&\hspace {-0.5pc}{\boldsymbol {\chi }_{\theta _{i}}={\hat {\boldsymbol {\theta }}}^{-}_{k}-\xi T \tanh \left [{\left ({\xi /\boldsymbol{\Upsilon }_{i,k|k-1}^{*}}\right)\left ({\mathbf {y}_{k}-\boldsymbol{\Upsilon }_{i,k|k-1}^{*}}\right)}\right]}. \\&\qquad\qquad\qquad\qquad\qquad\qquad\quad \displaystyle {{i=1, {\dots },2L+1}} \tag{35}\end{align*}

Before continuing to derivation for comparison, the behavior of the $\tanh $ function should be investigated. Maclaurin series of this function is

\begin{align*} {\tanh (x)=x-\dfrac {1}{3}x^{3}+\dfrac {2}{15}x^{5}-\dfrac {17}{315}x^{7}+\dfrac {62}{2835}x^{9}- {\dots },} \\ \tag{36}\end{align*}

and with Big O notation (36) can be expressed as

\begin{equation*} {\tanh (x)=x+\mathcal {O}(x^{2}).} \tag{37}\end{equation*}

In this notation, as $x$ close to zero the error of the series expansion is limited to a constant times $|x^{2}|$. In addition to that, this function is lower and upper bounded. Considering this information, (35) becomes

\begin{align*} \boldsymbol {\chi }_{\theta _{i}}=&{{\hat {\boldsymbol {\theta }}}^{-}_{k}- \left ({\xi ^{2}T./\boldsymbol{\Upsilon }_{i,k|k-1}^{*}}\right)\left ({\mathbf {y}_{k}-\boldsymbol{\Upsilon }_{i,k|k-1}^{*}}\right)} \\&-\,{\xi T\,\mathcal {O}\left ({\left [{\left ({\xi /\boldsymbol{\Upsilon }_{i,k|k-1}^{*}}\right)\left ({\mathbf {y}_{k}-\boldsymbol{\Upsilon }_{i,k|k-1}^{*}}\right)}\right]^{2}}\right).} \\&{i=1, {\dots },2L+1}\tag{38}\end{align*}

Obviously, the error term in (38) is limited to a constant times $\biggl |\left [{\left ({\xi /\boldsymbol{\Upsilon }_{i,k|k-1}^{*}}\right)\left ({\mathbf {y}_{k}-\boldsymbol{\Upsilon }_{i,k|k-1}^{*}}\right)}\right]^{2}\biggr |$ as $\boldsymbol{\Upsilon }_{i,k|k-1}^{*}\rightarrow \mathbf {y}_{k}$. Thus, by neglecting this error term another parameter update rule can be written as

\begin{align*}&\hspace {-0.5pc}\boldsymbol {\chi }_{\theta _{i}}= {{\hat {\boldsymbol {\theta }}}^{-}_{k}- \left ({\xi ^{2}T./\boldsymbol{\Upsilon }_{i,k|k-1}^{*}}\right)\left ({\mathbf {y}_{k}-\boldsymbol{\Upsilon }_{i,k|k-1}^{*}}\right).} \\&\qquad\qquad\qquad\qquad\qquad\qquad\quad\; \displaystyle {{i=1, {\dots },2L+1}} \tag{39}\end{align*}

If (32) provides a MAP parameter estimate with a Gaussian distribution assumption, so does the rule in (39) with a variable $\mathbf {R}_{\tilde {\mathbf {e}}}$ and measurement estimate dependent Kalman gain $\mathbf {K}=\xi ^{2}T./\boldsymbol{\Upsilon }_{i,k|k-1}^{*}$. It should be noted that the main difference between parameter update rules is the neglection of higher order terms in Maclaurin series expansion of the $\tanh $ function in (39). Therefore, parameter $\xi $ must be selected differently for the rule given in (39). To achieve same level of effective observation noise covariance $\mathbf {R}_{\tilde {e}}$, $\xi $ in (39) should be selected higher than it is in (32) since a square of this small parameter appears in Kalman gain. Nevertheless, with same selection of $\xi $, the convergence of the ModJUKF with (39) will be slower. A comparison of two parameter update rules are provided in the experimental results (Fig. 6).

**FIGURE 3.**
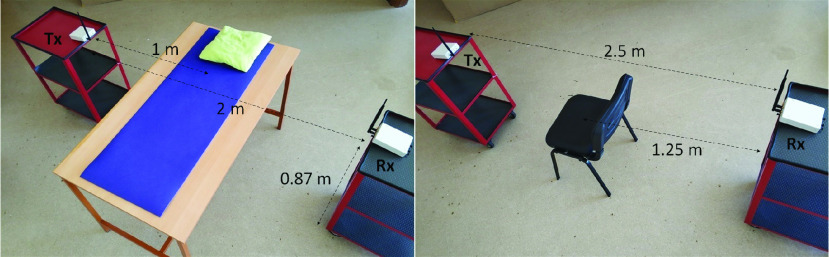
The real measurements are collected using the experimental setups in laboratory [22].

**FIGURE 4.**
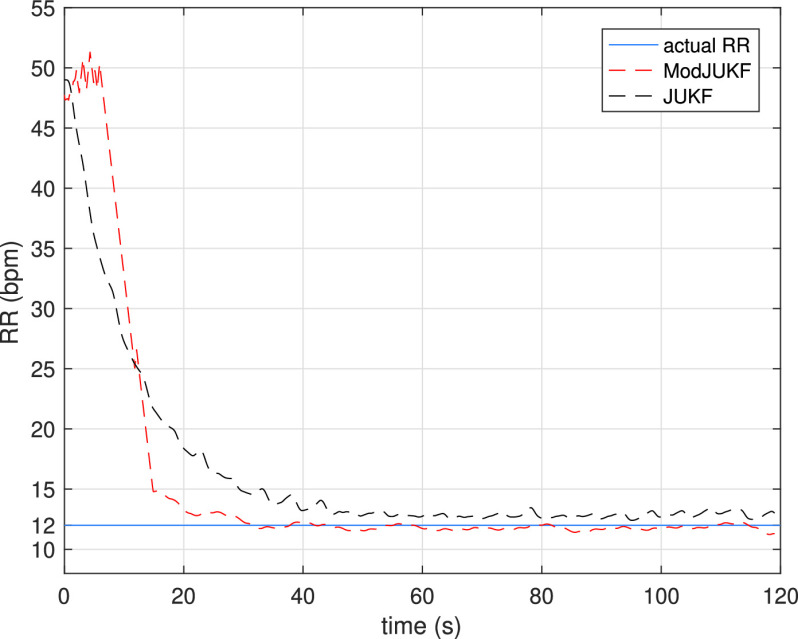
Respiratory rate (RR) tracking performances of the ModJUKF and JUKF methods in the constant RR scenario.

**FIGURE 5.**
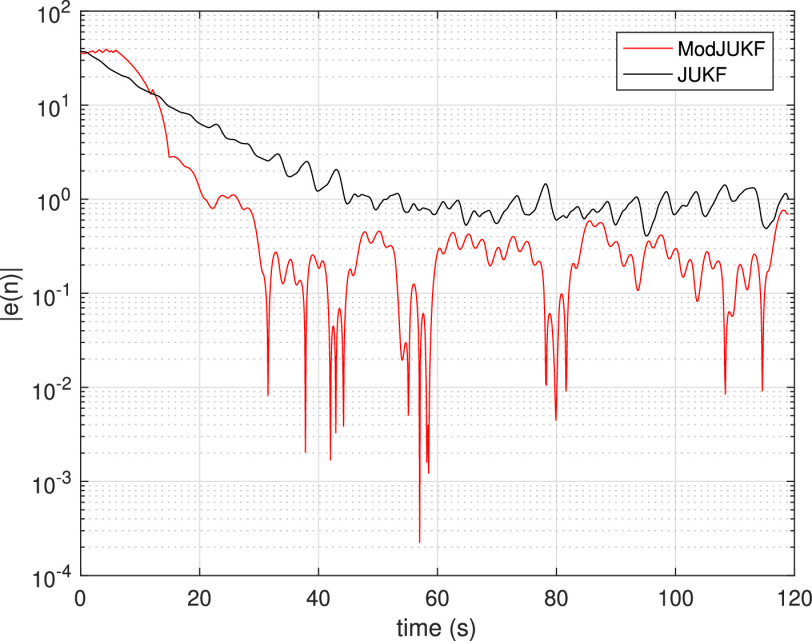
Absolute estimation errors of ModJUKF and JUKF methods in the constant RR scenario.

**FIGURE 6.**
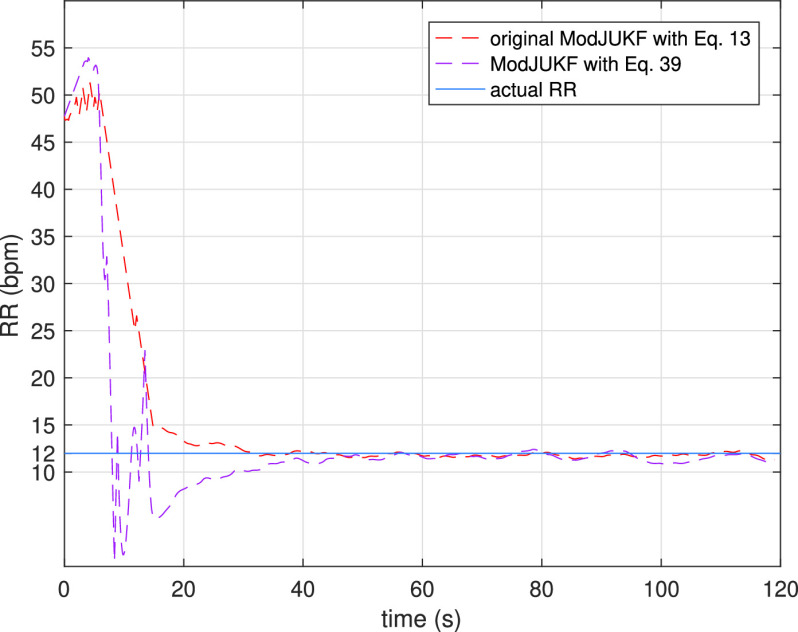
The RR estimation performance comparison for parameter update rules of ModJUKF.

In ModJUKF, the estimated parameters may have some undesired fluctuations around the true rate, which reduces overall accuracy. These fluctuations can be reduced by smoothing methods such as the $\alpha $-trimmed mean filter, the moving average filter, and the standard median filter [11] but they all require batch-processing as they use the estimates in a time window. Instead, for the smoothing of the RR estimates, we use the exponential filter which does not require batch-processing as it uses only one past output value. Thus, it is able to be applied to real-time applications. In the simple moving average filtering, the past output values are equally weighted, while the exponential filter uses the exponentially changing weights for the outputs. The exponential filter is as,

\begin{equation*} s_{k}=\gamma e_{k}+(1-\gamma)s_{k-1}, \quad k>0 \tag{40}\end{equation*}

where $e_{k}$ denotes the respiratory rate estimates, $s_{k}$ is the smoothed data. $\gamma $ is smoothing factor where $0 < \gamma < 1$. This smoothing process causes a small latency due to the cumulative structure of the filter, which increases the convergence time. To accelerate convergence, the smoothing process begins at $15^{th}$ seconds. The values of the smoothed data before $15^{th}$ seconds are selected the same as the estimates.

## IV. Results and Discussion

### A. Experimental Setup

The real measurements are taken using the two experimental setups which are shown in Fig. 3. Two Universal Software Radio Peripheral (USRP) B210 modules which are deployed two sides of a bed (or a chair) are used as the radio transmitter and the receiver. The radio transmitter emits a continuous wave carrier signal at 900 MHz. The transmit power of signal is adjusted as zero decibel-milliwatt (dBm). The receiver module down-samples and digitizes the RF signal to baseband for processing. After down-sampling, the sampling rate is specified as 10 Hz which ensures the Nyquist rate criterion for obtaining the respiratory frequency. Omni-directional antennas are used in both transmitter and receiver.

### B. Evaluation of Respiratory Rate Estimation

We design some experiments to obtain the real-time performances of the proposed ModJUKF method. The respiratory rate tracking performances of the ModJUKF method for time-varying and constant respiratory rate scenarios are given and compared with the standard JUKF method and high resolution ESPRIT and MUSIC approaches and the periodogram method. The real measurements are taken from 8 healthy participants. The participants breathe with rates in the range of 12 bpm (breath per minute) to 20 bpm in different experiments and they synchronize their respiratory rates with the help of a metronome which acts as a ground-truth. The absolute estimation errors of the ModJUKF and the JUKF for the constant RR scenario are also shown. The absolute error equals to $|e(n)|=60 \times |\hat {f}_{R}(n)-f_{R}(n)|$ in terms of bpm, where $\hat {f}_{R}$ and $f_{R}$ are the estimated and the actual respiratory rates in terms of Hz, respectively, and $n$ is the estimation index. The underlying reason for multiplying by 60 is to convert the frequency unit from Hz to breath per minute (bpm). The parameters $p$ in (9) and $\gamma $ in (40) are selected 0.9995 and 0.0093, respectively. These values are obtained empirically. In addition, state covariance matrix used in the experiments is taken as,

\begin{equation*} {\mathbf {Q}= \text {diag}~[10^{-10} \quad 10^{-10}]}\tag{41}\end{equation*}

and measurement noise covariance, $R$, is selected as 0.1. $\mathbf {Q}$ and $R$ are determined by physical intuition and empirically. Besides, the root mean square error (RMSE) values of all the methods for the time-varying RR scenario are illustrated. RMSE is calculated as follows,

\begin{equation*} RMSE~(bpm) = \sqrt {\frac {1}{N}\sum _{n=1}^{N}e(n)^{2}},\tag{42}\end{equation*}

where $N$ is the total number of estimates.

#### 1) Constant RR Scenario

In the first experiment, the behaviors of the Kalman Filter approaches in the constant respiratory rate scenario are investigated. In this scenario, the participant keeps his/her respiratory rate constant with the help of a metronome and does not change the rate during the measurements. Fig. 4 shows the RR tracking performance of the ModJUKF and the JUKF methods when the participant breathes at a rate of 12 bpm. As is seen, both methods converge to the actual rate, while the proposed ModJUKF method has less oscillation than the JUKF method, which increases accuracy. The absolute estimation errors of the methods for this measurement are shown in Fig. 5. It is also seen that the steady-state errors of the proposed method are less than 0.5 bpm. The error rate of the JUKF method, which has bias estimates, is around 1 bpm. Besides, the proposed ModJUKF method converges faster than the JUKF method as shown in the figure. In addition, two parameter update rules, which are given in (13) and (39), of ModJUKF are compared in Fig. 6. $\xi $ parameter, which is a scaling parameter, is chosen as 0.025 for the first rule in (13). As we discussed earlier, $\xi $ must be chosen higher than the first rule in (13) to achieve same level of observation noise covariance. Therefore, $\xi $ is chosen as 0.095 for the rule in (39). The selection of $\xi $ in (39) affects the convergence time of ModJUKF. It is seen that similar RR estimation performances are achieved by using both parameter update rules of ModJUKF.

**FIGURE 7.**
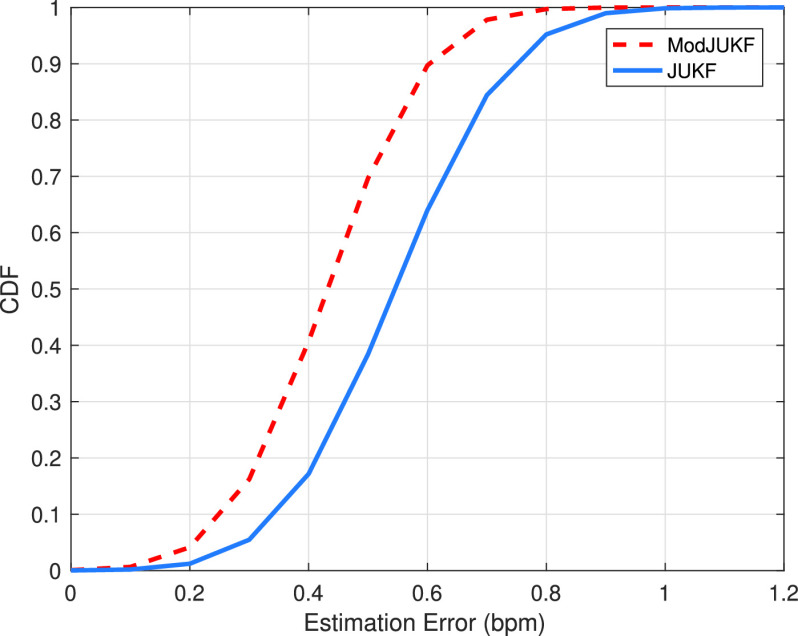
The CDF of the respiratory rate estimation error.

Fig. 7 shows the cumulative density function (CDF) of the respiratory rate estimation error for the proposed ModJUKF and the standard JUKF methods. The results are obtained by using a data set, which includes a total of 25 measurements, each 2 minutes long, collected from 8 healthy participants who breathe in a controlled manner for different respiratory rates in a range between 12 bpm and 18 bpm. As shown in the figure, over 90% estimation errors of the proposed ModJUKF method are less than 0.6 bpm which is a reasonable value for the real-time RR tracking. It can clearly be stated that the proposed ModJUKF method has superior performance than the standard JUKF.

#### 2) Time-Varying RR Scenario

**FIGURE 8.**
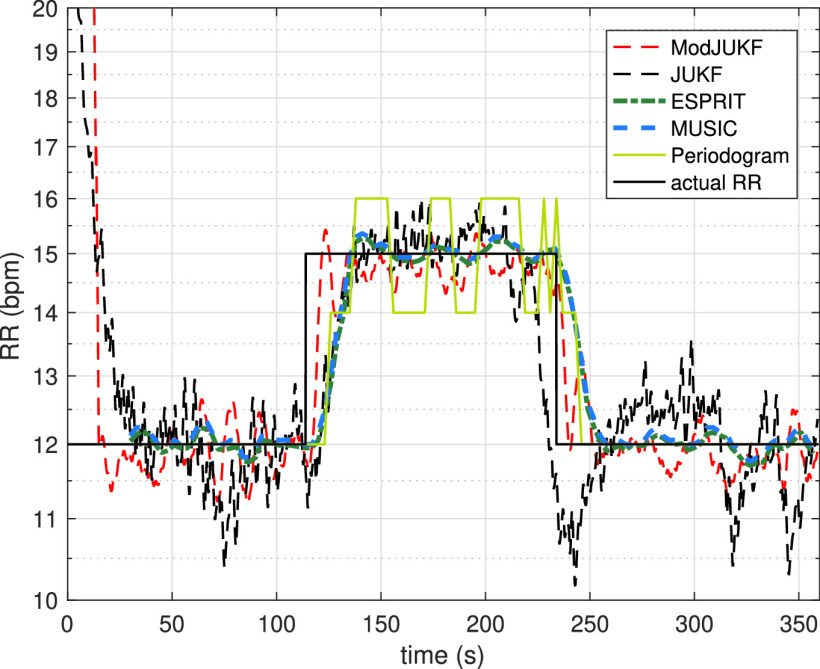
RR tracking performances of the methods in the time-varying RR scenario.

**FIGURE 9.**
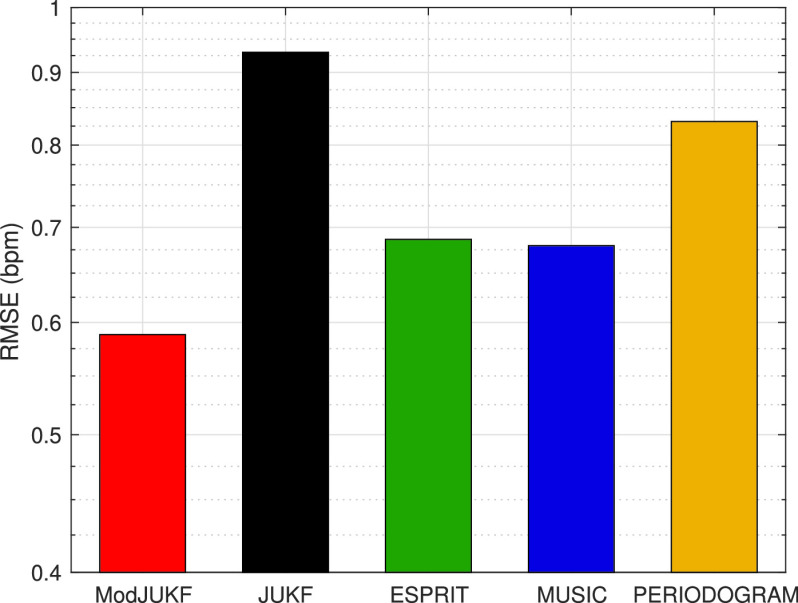
The RMSE values of the methods for the time-varying RR scenario.

In the second experiment, the case of time-varying respiratory rates is investigated. In the experiment, the participant changes his/her respiratory rate from 12 bpm to 15 bpm at ${114^{th}}$ seconds and from 15 bpm to 12 bpm at ${234^{th}}$ seconds in a controlled manner to observe the response of the methods to the sudden changes of the respiratory rates. The proposed ModJUKF method is tested according to this scenario and the standard JUKF method is also given for the comparison. The results for a 6-minutes recording are shown in Fig. 8. Although the proposed ModJUKF method has more oscillations according to the constant RR scenario, the proposed method can track the respiratory rate without missing the sudden changes after the convergence as seen in the figure. However, the standard JUKF method has considerable oscillations on the actual rate, which decreases accuracy. In addition, the RR tracking performances of the windowing-based methods such as high resolution ESPRIT and MUSIC methods and the periodogram method are given. As seen in the figure, these methods track the changing RR with latency due to the windowing approach. The window duration is selected as 30 s for these methods. The periodogram also has deviations, especially when the respiratory rate is 15 bpm, depending on the limited frequency resolution. The RMSE values of all the methods for this experiment are shown in Fig. 9. The proposed ModJUKF method has the lowest error among the other methods. High-resolution ESPRIT and MUSIC methods are expected to achieve very high accuracy in a constant RR scenario, while their accuracies decrease depending on the latency in the time-varying scenario. The JUKF shows the worst performance since it has many oscillations around the actual rate.

#### 3) Impact of Distance Between Transmitter and Receiver

**FIGURE 10.**
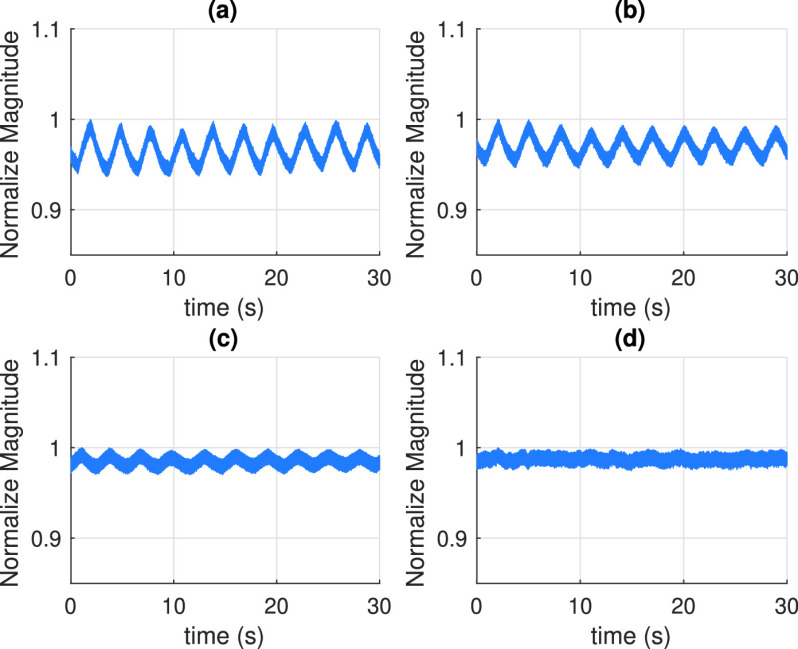
Received signal magnitudes according to distance between Tx and Rx: (a) 3m, (b) 4m, (c) 6m, (d) 8m.

**FIGURE 11.**
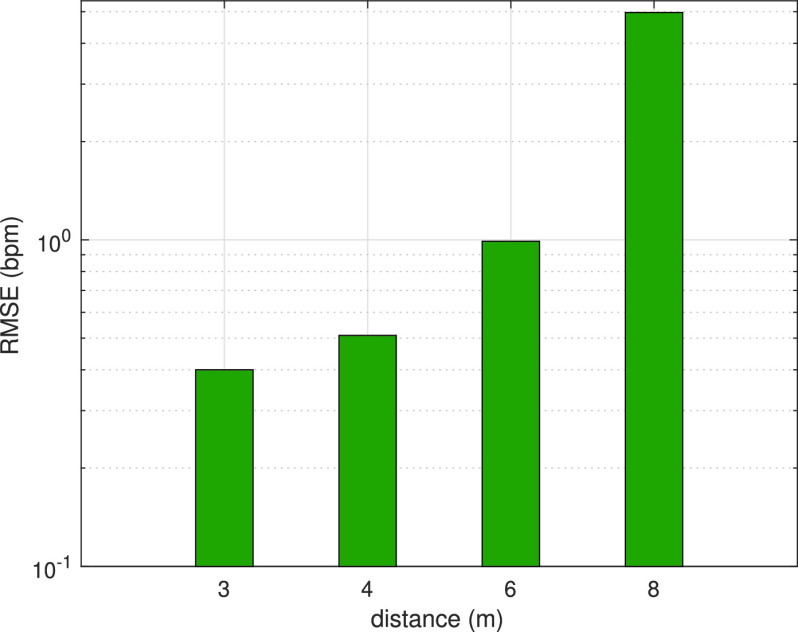
RR estimation errors (in terms of RMSE) of ModJUKF according to distance.

In the third experiment, the effect of changing the distance between the transmitter and the receiver on the performance is investigated. Measurements are taken for distances in the range of 3 to 8 meters. Fig. 10 shows the received signal magnitudes for these different distances. As seen in the figure, the effect of the chest movements caused by breathing on the received signal magnitude decreases with the increase of the distance between the transmitter and the receiver. When the distance reaches 8 meters, the effect on the signal disappears completely as shown in Fig. 10(d). Fig. 11 shows the respiratory rate estimation error values of the proposed method according to the distance. A decrease in performance is seen with the increase of the distance as shown in the figure. The strength of the radio signals that propagate in the air changes inversely with the distance. Therefore, the radio signals attenuate as they travel in free space. While error values are low both in 3 and 4 meters of distance, error increases when the distance is increased to 6 meters, but it is still in an acceptable range. When the distance reaches to 8 meters, the attenuation in the signal increases a lot and the signal drops below the noise level. Therefore, it can be said that it is hard to extract the breathing signal to estimate the respiratory rate in 8 meters.

#### 4) Impact of Human Orientation

**FIGURE 12.**
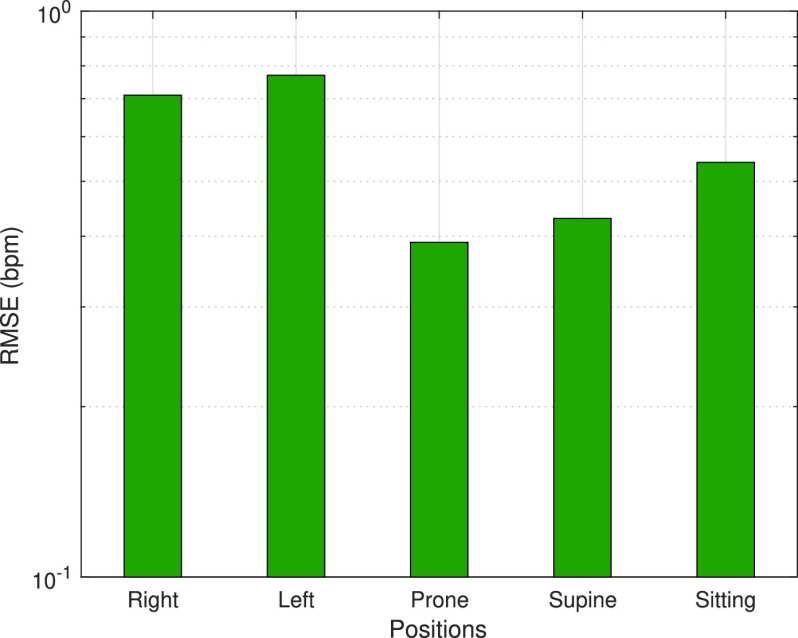
RR estimation errors (in terms of RMSE) of ModJUKF according to human orientation.

Fig. 12 shows the effect of the orientation of the human to the RR estimation performance. The purpose of this experiment is to reveal how human orientation affects RR estimation. To test this case, five different human positions are taken into account, four of which are lying positions and one is sitting position. In lying positions, the participants lie on the bed in the experimental setup shown in Fig. 3(a) in the supine, prone, and right and left side positions. Better performances are obtained in prone and supine positions since the number of active paths in these positions is more than the paths in the one-side lying positions. The active path is defined as the one that is more affected by human breathing than other paths [13]. In addition, the participants sit on the chair as shown in Fig. 3(b) for testing the sitting position. It can be seen that the proposed method attains 0.53 bpm RMS error in this position. It is clearly seen that our proposed system can accurately estimate RR without being affected by human orientation.

#### 5) Impact of Different Environments

**FIGURE 13.**
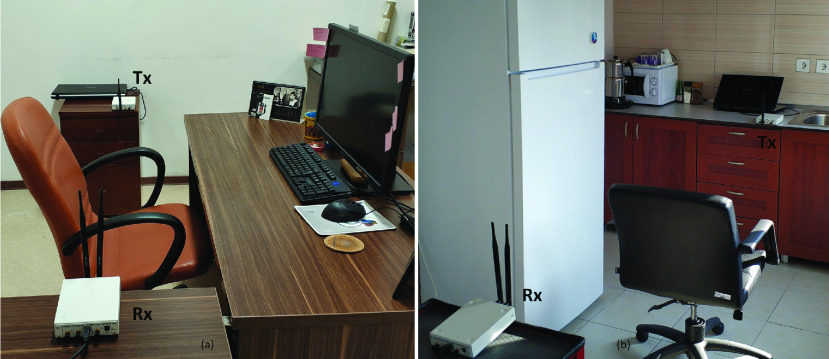
Test environments: (a) Office, (b) Living Area [22].

**FIGURE 14.**
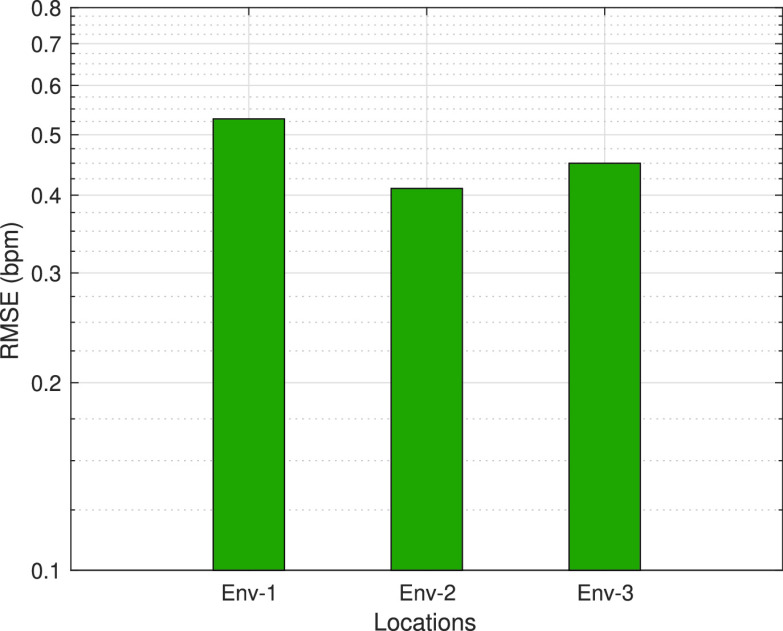
RR estimation errors (in terms of RMSE) of ModJUKF according to environments (Env-1: Laboratory, Env-2: Office, Env-3: Living Area).

In this experiment, we applied the proposed RR estimation method for the RF signals which are collected in different places to see the effects of the environments. RF signals are easily affected by the environmental conditions. Especially indoors, the amplitude of the RF signal can change due to some effects such as reflection, scattering, etc. The size of the room, the furniture in the room, even the number of WiFi signals as the interference source are different in each environment. Hence, the purpose of setting up experiments in different places is to illustrate that the proposed RR monitoring system is minimally affected by these changing factors. The proposed method is tested in two different places in addition to the laboratory with this goal. Environment-1 is the laboratory shown in Fig. 3, where all other experiments are carried out. Environment-2 is an office room in the university and Environment-3 is a living area with a kitchen inside, which are shown in Fig. 13. RR estimation performances of the proposed method for these environments are shown in Fig. 14. Performances are obtained using 45 minutes of measurements. The proposed method can accurately estimate RR in all three environments according to the performances in the figure. It is demonstrated by this experiment that the proposed method can estimate RR regardless of the environmental changes and achieve high accuracy in different environments.

#### 6) Impact of Breathing Forms

**FIGURE 15.**
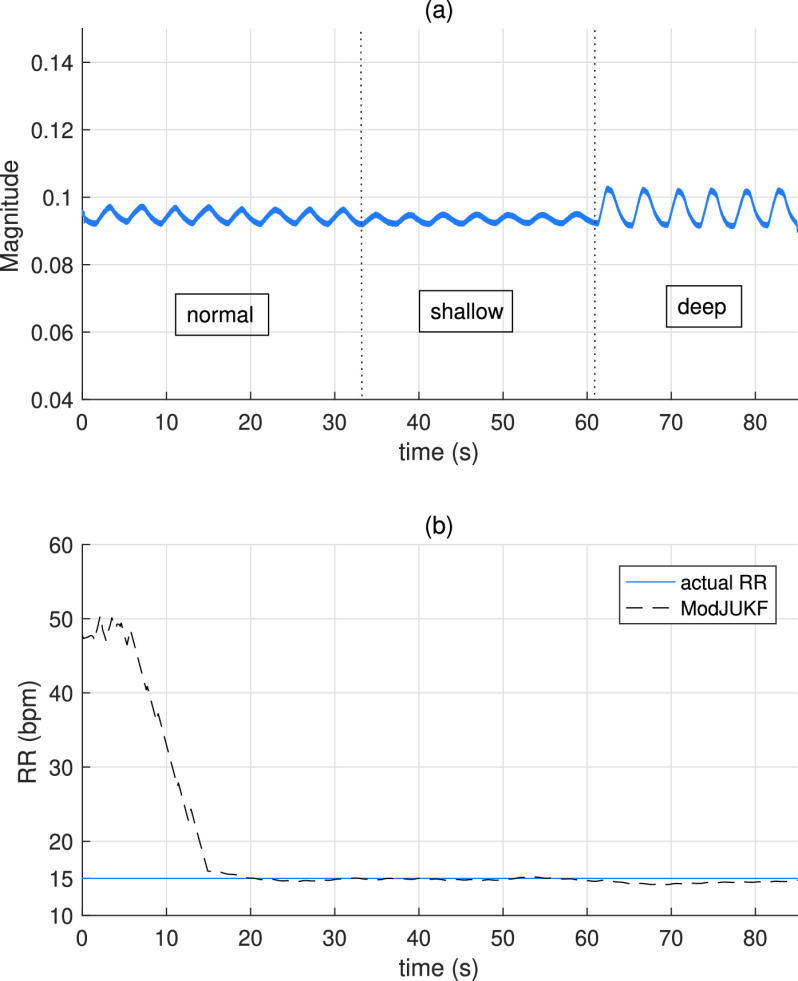
(a) Received signal magnitude during different breathing forms. (b) RR tracking performance of ModJUKF for different breathing forms.

Even the respiratory rate is same, breathing forms can vary from person to person. A robust RR estimation method should not be affected by these variations. In this experiment, the proposed method is tested for different breathing forms. Fig. 15(a) shows the received signal magnitude for different breathing forms. The participant who lies on the bed breathes normally during the first 30 seconds. Then, he takes breaths shallowly in the next 30 seconds, and finally, he takes deep breaths until the experiment lasts. As seen, peak-to-peak values of received signal change according to the breathing form. While chest displacement is minimal in shadow breathing, the person draws more air into the lungs in deep breathing form. Fig. 15(b) shows the RR estimation performance of the proposed method for this scenario. As seen in the figure, the proposed method can track RR accurately after convergence regardless of breathing forms.

#### 7) Computational Complexity Comparison

Lastly, computational complexities of JUKF and ModJUKF are compared. Stopwatch timer in MATLAB^®^ 2016a software is considered for comparison. Same coding style is used for both JUKF and ModJUKF, and codes are run on a ASUS ROG CG8580 desktop computer with a 4.60 GHz Intel Core™ i7-3770K processor and 24 GB RAM. 6 minutes of recording, for which the results are illustrated in Fig. 8, is used for comparison. Time step considered in the realization of mathematical models is same for both JUKF and ModJUKF, and it is 0.1 s. Codes are run 10^3^ times for both JUKF and ModJUKF, and the average elapsed times are recorded. Average elapsed time for JUKF is found as 0.3905 s, whereas it is 0.3594 s for ModJUKF. It can be seen that ModJUKF reduces the computational complexity approximately 8.54% with respect to the JUKF. In this parameter estimation case, actually the number of states is 2 in ModJUKF and 3 for JUKF as provided in (3) and (4), but the last state is used for parameter estimation in JUKF. As previously stated, the number of reduction in sigma points for ModJUKF is $\frac {(2L_{\theta })}{(2(L+L_{\theta })+1)}\times 100 = \frac {2\times 1}{(2(2+1)+1)}\times 100=28.5\%$. However, due to the separate parameter update rule given in (13) and (14), which include the calculation of a unary function $\tanh $ and a mean operation, the real reduction in computational complexity is found as ≈ 8.54%. Clearly, ModJUKF is a promising alternative to JUKF for respiratory rate estimation with respect to its accuracy and computational complexity.

## V. Conclusion

Nowadays, the potentials of ambient wireless radio signals are investigated for fall detection, elderly health monitoring etc. for home-care applications. In our recent study, a non-contact RR monitoring system with a high accuracy subspace estimation method is presented which uses low-cost software-defined radios. It is observed that in the case of sudden change in RR, the windowed methods can not suddenly track the changes. The RR estimation latency is proportional to the window size which means tens of seconds.

In this paper, a new real-time non-contact RR estimation and tracking algorithm is proposed. Briefly, the standard joint unscented Kalman filter method is modified for the transformation of the measurement error into parameter error by using the hyperbolic tangent function. The proposed method is validated by the various experiments compatible with realistic scenarios to show the practical factors affecting RR estimation performance. It is shown in the experiments conducted with real measurements that the proposed ModJUKF method offers both a decrease in computational complexity of 8.54% and an increase in accuracy of 36.7% according to the JUKF method. It is also shown that the proposed ModJUKF method outperforms the windowing-based methods in the time-varying RR scenario.
